# Managing a Complicated Acute Otomastoiditis at Day Four of Life

**DOI:** 10.7759/cureus.12905

**Published:** 2021-01-25

**Authors:** Rachel Lim, Shifa Zulkifli, Iskandar Hailani, Noor Dina Hashim

**Affiliations:** 1 Otolaryngology - Head and Neck Surgery, Universiti Kebangsaan Malaysia Medical Centre, Kuala Lumpur, MYS; 2 Otolaryngology - Head and Neck Surgery, Hospital Kuala Lumpur, Kuala Lumpur, MYS; 3 Otorhinolaryngology - Head and Neck Surgery, Hospital Tunku Azizah (Women and Children’s Hospital) Kuala Lumpur, Kuala Lumpur, MYS

**Keywords:** acute mastoiditis, otomastoiditis, facial nerve palsy, newborn, chronic granulomatous disease

## Abstract

Acute mastoiditis in a newborn complicated by the presence of facial nerve palsy is an alarming finding requiring rapid assessment and further investigation. Such an early presentation should point the clinician towards an underlying systemic pathology or congenital anatomical abnormality. Facial nerve involvement indicates severe infection and possible dehiscence of the facial canal. Although more frequent in children, it is rare in neonates. We would like to share our experience in managing the youngest known presentation of otomastoiditis at four days of life. The patient presented with otorrhea and facial paralysis and progressed to meningitis. He was finally diagnosed with chronic granulomatous disease.

## Introduction

Acute mastoiditis is a serious disease and the most common sequelae of acute otitis media. Acute otitis media frequently affects children less than two years of age [1]. The incidence of this illness has dramatically reduced after the introduction of antibiotics [1]. Infants are unique as in this age group the middle ear anatomic characteristics develop in a few stages. The mastoid antrum in this age group is connected by a narrow channel to the tympanic cavum where an inflammatory process may create an attic-tympanic membrane causing separation of the aditus ad antrum and tympanic cavum [1]. Thus, the secretions remain within the antrum and the infection spreads by eroding the mastoid cortex or by extension into the emissary veins of the mastoid bone causing osteitis [1]. Rarely, it progresses to life-threatening complications like meningitis, brain abscess, and venous thrombosis, as observed in this case. These intracranial complications occur due to the spread of infection from the mastoid cavity via bony dehiscences or erosions of the tegmen with osteitis, unossified petrosquamous suture in children, or thrombophlebitis of small veins communicating with the lateral sinus [2].

## Case presentation

A four-day-old male infant presented acutely with right ear hemoserous discharge and right facial asymmetry for one day. The child was noted to be less active with poor feeding. There were no fevers or respiratory symptoms. He was born term at 38-week gestation, breast-fed, and was from a non-consanguineous marriage. The delivery was uncomplicated and he had been discharged well on the second day of life.

**Figure 1:**
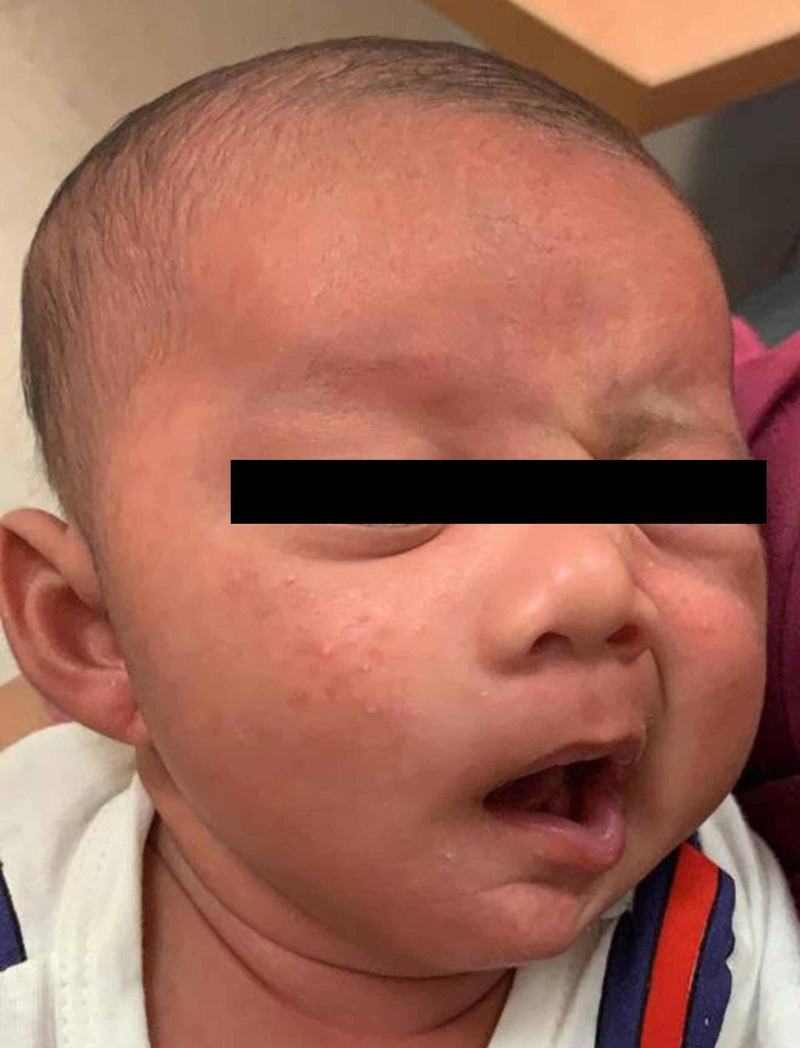
Patient with right facial nerve paralysis with incomplete closure of the right eye.

On examination, the child was lethargic and non-febrile. There was House-Brackmann grade IV right-sided facial nerve paralysis (Figure 1), with copious amount of right ear hemoserous discharge and edematous external auditory canal. Tympanic membrane could not be appreciated. There was no swelling or erythema over the postauricular area. Examination of the left ear was grossly normal. The eyes were noted to have minimal yellowish discharge.

**Figure 2:**
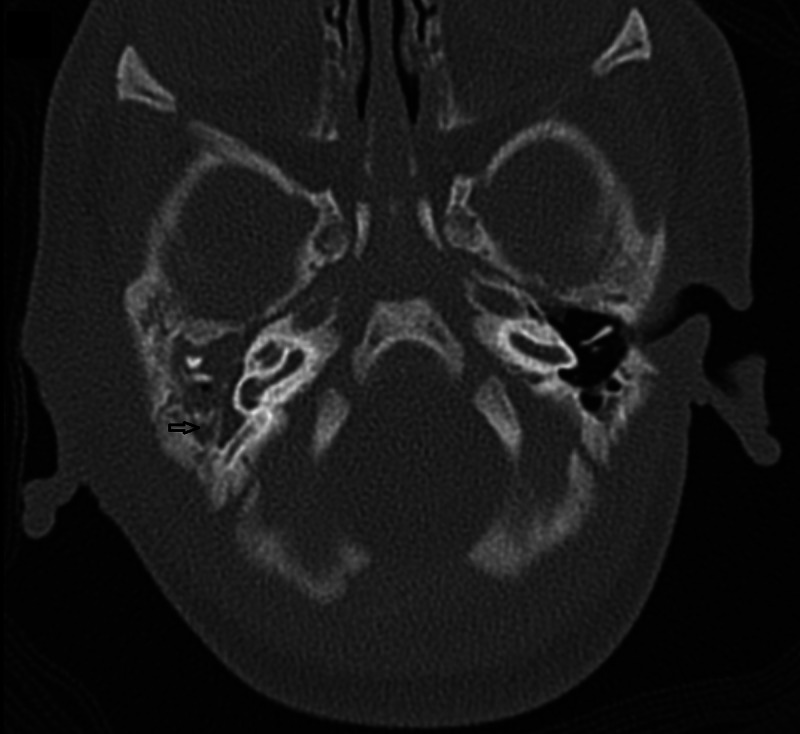
CT of the temporal bone (axial view) showing soft tissue and fluid in the middle ear with fluid-filled right mastoid air cells. The mastoid portion of the facial canal is dehiscent (arrow). The normal configuration of the ossicles could not be visualized. CT, computed tomography

Following admission, full septic workup was performed and broad-spectrum intravenous antibiotics were commenced. Blood investigations showed pancytopenia with an elevated C-reactive protein. A contrast high-resolution computed tomography (CT) scan of the temporal bone demonstrated a soft tissue density at the right external auditory canal extending into the middle ear. The right mastoid air cells and middle ear cavity were fluid-filled with dehiscence of the mastoid portion of the facial canal (Figure 2). The right jugular bulb and internal jugular vein were not opacified suggesting venous thrombosis. *Pseudomonas aeruginosa* bacteremia was identified from the blood culture. Ear swab cultures grew mixed growth while eye swab cultures showed infection with *P. aeruginosa* and *Staphylococcus aureus* spp. Despite antibiotic treatment, the child deteriorated and developed respiratory distress and seizures requiring intubation on day four of admission. Lumbar puncture revealed raised total protein content with low glucose levels in the cerebrospinal fluid suggesting bacterial meningitis. Infective screening for viral hepatitis, syphillis, and human immunodeficiency virus was unremarkable. Further tests including dihydrorhodamine test and lymphocyte surface marker analysis were suggestive of chronic granulomatous disease (CGD). Following this, a single dose of intravenous immunoglobulin (IVIG) (0.5 g/kg) was administered. Soon after, clinical improvement was observed and the child was extubated three days later. A subsequent multidisciplinary discussion had come to an agreement to optimize the child’s condition prior to embarking on any surgical intervention.

**Figure 3:**
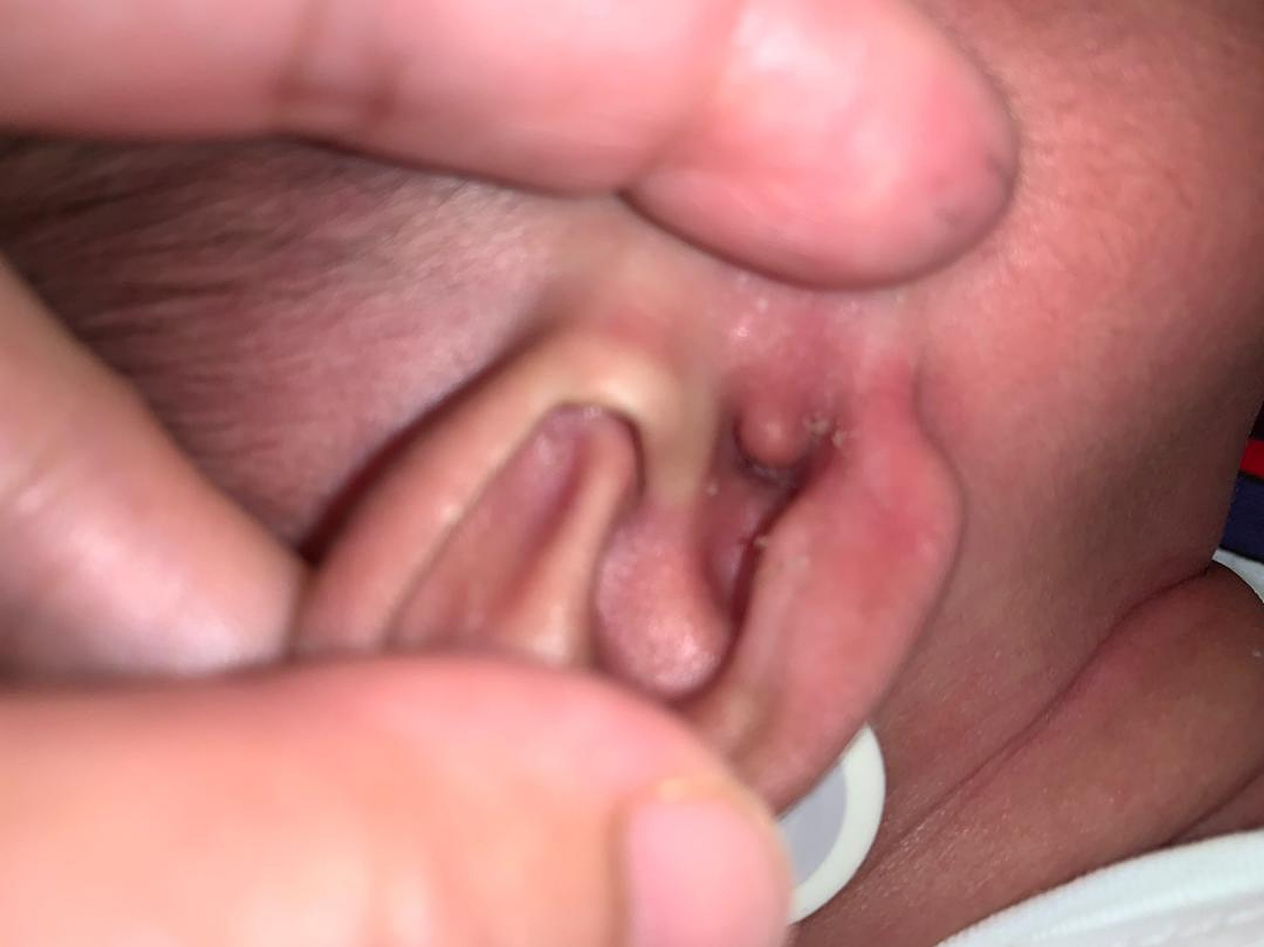
External auditory meatus completely occluded by polypoidal tissue.

After a six-week course of intravenous antibiotics consisting of the cephalosporin and aminoglycoside group, the otorrhea gradually resolved. Regular antibiotic eardrops were administered along with ear toileting. However, there was an ear polyp occluding the external auditory canal (Figure 3). Facial nerve function remained the same and auditory brainstem reflex confirmed a mild conductive hearing loss on the right with normal hearing on the left. Prophylactic trimethoprim-sulfamethoxazole (5 mg/kg/day) and itraconazole was initiated after completion of intravenous antibiotics and the child was discharged. Subsequently, he underwent right cortical mastoidectomy at three months of age. Intraoperatively, there was granulation tissue occupying the mastoid cavity with erosion of the ossicles and complete stenosis of the external auditory canal. Histopathologic analysis showed fibrous tissue with presence of spindle cells. These cells contain oval-to-spindle-shaped nuclei and scanty eosinophilic cytoplasm. There was no evidence of necrosis or malignancy and immunohistochemistry studies were unremarkable. Culture of the granulation tissue was negative and tuberculosis polymerase chain reaction was negative. A review two weeks post-operatively noted an improvement in facial nerve palsy to House-Brackmann grade II.

## Discussion

Acute otomastoiditis in infants may not present with the typical symptoms of otalgia, otorrhea, and reduced hearing seen in older children and adults. Infants often display non-specific symptoms such as fever, vomiting, diarrhea, and poor feeding [1]. This highlights the importance of a thorough examination in infants to uncover crucial signs, namely, mastoid tenderness or swelling, inflamed or bulging tympanic membrane, facial palsy, and otorrhea. The unusually rapid development of otomastoiditis in any newborn necessitates further investigation and consideration of an underlying immunological deficiency. In fact, our case is possibly the youngest otomastoiditis ever reported. Matsubara et al. reported a similar case of acute mastoiditis presenting at two months of age which was later diagnosed with severe congenital neutropenia while another case was reported by Parpounas et al. which presented at 28 days of age [3,4]. Sousa Menezes et al. published a similar case in an 11-day-old newborn complicated by subperiosteal abscess with underlying transient hypogammaglobulinemia [5].

CGD is an inherited immunodeficiency that affects one in 250,000 individuals [6]. It occurs secondary to a defect in the enzyme leukocyte NADPH oxidase which is involved in phagocytosis. Thus, myeloid cells are rendered incapable of phagocytosis resulting in severe recurrent infections. The main systems involved in CGD are the lungs followed by the skin, lymph nodes, gastrointestinal system, and liver, which was not present in this case. In a study of 429 patients with CGD, otitis media was observed in 14%, with *P. aeruginosa* being the most common identifiable organism [6]. Although the ear swab cultures in our case had mixed growth, likely due to contamination, the blood cultures grew *P. aeruginosa*, which we suspected had originated from the primary site of infection which was the ear.

The facial nerve involvement in our case can be attributed to the middle ear infection spreading to the fallopian canal through bony dehiscences. These dehiscences may be congenital or occur following erosion by infection [7]. Another possibility is the inflammation and edema of the nerve within the narrow bony canal with accumulation of purulent material causing compressive ischemic neuritis [8]. Some also hypothesize that the bacterial toxins produced cause demyelination of the facial nerve [7]. CT scan is useful to assess the facial canal; however, bony dehiscences may not always be detected, especially in such a small temporal bone.

Otomastoiditis complicated by facial nerve palsy warrants admission and intravenous antibiotics with blood-brain barrier penetration. In this antibiotic era, acute mastoiditis is primarily managed medically as studies have shown antibiotics to be effective in treating uncomplicated cases of mastoiditis [9,10]. Nevertheless, medical treatment alone may not be sufficient as antibiotics have lower bone penetrance compared to soft tissue and can result in residual diseased bony tissue [9]. Most authors recommend myringotomy to allow drainage and prevent accumulation of purulent material as well as to obtain proper microbiological specimens [7]. Mastoidectomy is indicated in patients with intracranial complications and poor clinical improvement despite adequate antibiotic therapy [9,10]. Baljosevic et al. recommended that mastoidectomy be done if an infant who fails to respond after 10 days of antibiotic treatment [1]. However, it should be noted that their study population only included infants three months and older with no immunodeficiency. Decompression of the facial nerve has been suggested by Gaio et al. if the infant demonstrates >95% facial nerve degeneration on electroneuronography performed 3-14 days after the onset of nerve paralysis [7].

In an infant with suspected immunodeficiency, the decision for mastoidectomy should be discussed with the pediatrician and its risks and benefits weighed. A similar case was reported by Chang et al. involving a nine-year-old with CGD who underwent mastoidectomy complicated with delayed wound healing [11]. Delayed wound healing in patients with immunodeficiency is common and may lead to fistula formation [11,12].

In this patient, the timing for mastoidectomy was delayed due to the derangements in the blood parameters with ongoing sepsis. As blood volume in a newborn is proportionate to the weight, even a small amount of bleeding constitutes a significant proportion of the circulating blood volume. In addition, the underdeveloped temporal bone in a newborn with ongoing inflammation poses a challenge in identification of the facial nerve and the surrounding structures. The child also had responded to treatment with antibiotics and IVIG with improvement of the septic parameters. However, in view of the persistent facial nerve palsy and residual polyp causing occlusion of the external ear, mastoidectomy was indicated despite the known complications of poor wound healing.

Our patient was started on lifelong prophylactic antibiotics which is the mainstay of treatment in CGD. There is substantial evidence showing significant reduction in infections in patients who are given prophylactic antibiotics, namely, trimethoprim-sulfamethoxazole and itraconazole [6,13]. The role of IVIG has been demonstrated in a large randomized controlled study that compared prophylactic treatment with IVIG versus placebo and proved IVIG to be effective in reducing the risk of infection by 67% [14]. However, there has yet to be a consensus on the routine administration of IVIG as a prophylactic or therapeutic treatment modality as well as to the duration of treatment.

## Conclusions

A complicated case of acute otomastoiditis in a newborn is exceptionally rare warranting investigation for an underlying immunodeficiency. A high degree of suspicion coupled with rapid assessment and workup with initiation of antibiotics is paramount as the clinical condition tends to deteriorate quickly. Management should include both medical and surgical intervention to address the immunodeficiency and to remove of the foci of infection.
